# Learning to mark: a qualitative study of the experiences and concerns of medical markers

**DOI:** 10.1186/1472-6920-6-25

**Published:** 2006-04-25

**Authors:** Kamila Hawthorne, Fiona Wood, Kerenza Hood, Rebecca Cannings-John, Helen Houston

**Affiliations:** 1 Department of General Practice, School of Medicine, Cardiff University, Third floor, Neuadd Meirionnydd, Heath Park, Cardiff CF14 4XN, UK

## Abstract

**Background:**

Although there is published research on the methods markers use in marking various types of assessment, there is relatively little information on the processes markers use in approaching a marking exercise. This qualitative paper describes the preparation and experiences of general practice (GP) teachers who undertake marking a written assessment in an undergraduate medical course.

**Methods:**

Semi-structured interviews were conducted with seven of the 16 GP tutors on an undergraduate course. The purposive sample comprised two new markers, two who had marked for a couple of years and three experienced markers. Each respondent was interviewed twice, once following a formative assessment of a written case study, and again after a summative assessment. All interviews were audio-taped and analysed for emerging themes. A respondent validation exercise was conducted with all 16 GP tutors.

**Results:**

Markers had internal concerns about their ability to mark fairly and made considerable efforts to calibrate their marking. They needed guidance and coaching when marking for the first time and adopted a variety of marking styles, reaching a decision through a number of routes. Dealing with pass/fail borderline scripts and the consequences of the mark on the student were particular concerns. Even experienced markers felt the need to calibrate their marks both internally and externally

**Conclusion:**

Previous experience of marking appears to improve markers' confidence and is a factor in determining the role which markers adopt. Confidence can be improved by giving clear instructions, along with examples of marking. The authors propose that one method of providing this support and coaching could be by a process of peer review of a selection of papers prior to the main marking. New markers in particular would benefit from further guidance, however they are influenced by others early on in their marking career and course organisers should be mindful of this when arranging double marking.

## Background

Within medical education considerable attention has been devoted to the development of teaching methods, but examiners are often expected to know how to mark assessments, being regarded as experts in their field. Marking written essays is considered particularly challenging in that it requires the marker to apply more subjective measures of quality, often resulting in differences of opinion. The fact that these differences exist indicates that scorers are thinking differently about the content and process upon which scoring is based. Previous research has described techniques using a 'think aloud' task to describe marking activity during the marking process itself [1,2]. Their results help us understand the ways essay scorers read and mark papers, and describes differences between scorers, but do not investigate their ideas, concerns and expectations in preparing and marking itself. It was considered that the most appropriate way to explore the contextual issues of marking subjective papers, was to adopt a qualitative approach. These issues are explored in this paper, which describes the use of semi-structured interviews to get markers to reflect on their methods and approaches to the marking process.

The example used here is an assessment involving the marking of a Primary Care case based essay, an assignment where stringent attempts have previously been made to ensure standardisation of instructions to students and markers and the provision of a detailed marking schedule (see [3] for details of the marking schedule). (Usually concerns about reliability are dealt with by tightening up the marking schedule, but in this case this process had already taken place, and we wanted to take the investigation of the concept of decision making a step further.) More specifically, it aims to provide insights into markers' decision making and internal negotiating strategies, especially in cases where difficult decisions needed to be made. The authors also uncover learning points that can help markers of all experiences to develop their marking skills.

## Methods

Being a small scale exploratory study in an area where there is little published, qualitative methods were selected as being most appropriate. The underlying theoretical framework assumes an 'etic' stance, (ie using an outside observer of informal, semi-structured discussions who moves from an understanding of markers' perspectives to analyse and gain helpful insights into the internal processes that take place in making marking decisions).

We were anxious not to impose our own preconceptions, so an open approach was adopted using semi-structured interviews to afford greater flexibility for the interviewer and opportunity for the participants to reflect. Special attention was paid to ensuring multiple coding of the data, with team-based discussions so that all themes being developed were not relying on one coder alone, who could miss or misinterpret lines of discussion. Data from the first set of interviews (conducted after the markers had marked a set of formatively marked cases) was used iteratively to modify the approach and structure of the second set of interviews (after the marking of a set of summatively marked cases four months later).

The focus for the interviews was an assessment involving a written case presentation prepared by third year medical students coming to the end of their General Practice attachment at the School of Medicine, Cardiff University. The case presentation aims to assess students' ability to construct a diagnostic synthesis of an acute case they have seen whilst on attachment. It is formatively marked by their tutors in January and a second case is summatively marked in early May by a tutor unknown to them.

The assessments are marked by one of sixteen General Practice (GP) tutors, who have participated in regular workshops to discuss their teaching and the development of the marking schedule with senior academic support. The tutors have a range of experience as clinical teachers, from new markers to those with up to 7 years marking experience. Each tutor marks 15–20 case presentations both in the formative and the summative assessments. They are encouraged to give written feedback to individual students in both assessments. A sample of scripts are double marked independently and then discussed.

Semi-structured face-to-face interviews were held with seven of the GP tutors, including one pilot interview. A purposeful sampling strategy was used so as to include respondents with a wide range of marking experience. The sample included two new markers, two markers with moderate experience (1–4 years) and three experienced markers (over 5 years experience). Markers were approached by the interviewer, following a letter to all the markers that explained the purpose of the study. There were only two new markers, and two with moderate experience. All four were approached to take part in the study, and all four agreed. The remaining three markers were chosen from a sample of 12, one of them worked geographically close to the department (and was picked for the pilot interview), the remaining two were chosen for their longevity of marking. Again, all experienced markers approached to take part, agreed to do so. Table 1 details the topics covered in the interviews.

We interviewed each respondent twice: once after the formative marking in January 2004, and again after the summative marking in May 2004. Each interview was audio-recorded, anonymised on transcription and imported into a qualitative data analysis software package (NUD.IST) which aids the management, coding and retrieval of qualitative data. Following familiarisation with the data, it was indexed so that each mention of a particular issue was identifiable and retrievable. This formed the basis of a thematic framework, which incorporated the main objectives and themes of the data [4,5], and covered the range of views expressed. Markers' experiences of marking were explored during the interviews, with discussion of interim data collected between the first and second interviews of markers to confirm findings, develop themes and decide on further lines of discussion. Analysis was grounded in the data collected from the first set of interviews, so that respondents guided the direction of the analysis. In the data analysis new markers were compared with experienced markers, looking for the ideas and concerns of markers, details of their methods of marking, the degree to which markers conferred or wished to confer with each other, and their training and support needs. Sections of data were coded according to the main themes which were subsequently refined into narrower categories (Table 2). The framework was applied to all the data collected, looking for areas of concordance and also inconsistencies. Each interview transcript was read and coded by more than one member of the research team, thereby ensuring rigorous comparison of coding of the same data by multiple researchers and allowing ambiguities in coding to be resolved by discussion amongst the researchers [6].

Early findings were presented to the group of tutors in September 2004, with the presentation and ensuing discussion being audio-recorded. Such feedback is a form of respondent validation and can serve to test emerging theories, gather new evidence and enhance the credibility of the research [7]. Feedback from this meeting indicated that the markers supported the research team's findings. In particular there was a useful discussion on the merits and problems of using 'gut-feelings' when marking.

## Results

The pilot interview did not identify any problems and therefore it was decided to include the pilot interview within the analysis in order to increase the sample size.

The results obtained fell into three main sections:

1. Markers' appreciation of the inherent subjectivity of the marking of 'essay' style papers, and their concerns to mark reliably.

2. The efforts they made individually to calibrate internally and make sure the conditions for marking were right.

3. Outside influences that affected either their method of marking, or their decision making processes in awarding marks. This included the influence of more 'experienced' markers and the implications of making the 'wrong' decision.

Themes within these sections are discussed below, and summarised in Table 2.

### Concerns about marking

In terms of internal reliability, markers worried about their consistency. They were aware that concentration may falter during prolonged marking sessions and were concerned to mark all assessments in the same frame of mind so as to not disadvantage some. They were also aware that a good case study followed by a poorer one could make it seem worse than it really was.

GP2: *I think it depends on the order you mark them in. Now I had one that was really outstanding. He'd gone to masses of effort, drawn diagrams and got all the ideas – and then of course the next one doesn't compare very well *.(new marker describing the 'halo' effect)

The markers also brought up issues of external reliability, that is, how their own marking compared to others. Those with any experience knew from previous exam board meetings that the group contained softer and harder markers or, as they put it, 'hawks and doves'. Many of the respondents reflected on their perceptions of their place along this spectrum.

GP1: *We don't want the students all to become the same student just in different bodies and we don't want us to become the same markers just in different bodies. So in other words those who are hawks, if I can put it that way, I'm happy with them to be hawks but I think its not the object of the exercise for them to regress towards me but for them to at least realise they are hawks. And for people like me who are slightly more dovish to at least realise that *.(experienced marker)

Objectivity was also considered to be a concern of markers. Tutors described typical pet hates (poor referencing, poor grammar, poor use of headings etc), but also more subtle issues of style. For example one moderately experienced marker (GP5) discussed how she had problems staying objective when she felt the student's tone throughout the assessment had been arrogant.

GP6: *I think if someone has written it in a way that really rubs me up the wrong way in the presentational style then perhaps I will subconsciously mark them down, and perhaps I struggle with whether I'm being fair *.(experienced marker)

Respondents felt good markers were disciplined, open to other presentational styles, decisive, constructive, knowledgeable of the subject and familiar with academic conventions. Experience was considered crucial to these qualities. New markers described how they felt insecure about their marking. They wanted other markers to give them feedback on their marking and the amount and type of comments they returned to students.

GP4: *That's why I feel I'm probably not the best of markers, I have so little experience of it and so little sort of feedback about my marking...So I wouldn't be too surprised if I wasn't a very good marker. Its difficult to know. Just have to accept you might be off beam *.(new marker)

GP2: *I don't want to do the summative ones having had no idea of whether I was pitching myself at the right place, pitching the marking at the right place because again that wouldn't be fair on the students *.(new marker)

In these instances new markers dealt with their inexperience by informally approaching more experienced markers to ask them to shadow mark. Although new markers felt insecure about their marking, by the second interview (after marking both the formative and summative assessments) they were expressing more confidence in their abilities. The formative assessment was valued by the newer markers for providing them with marking experience. However marking the formative assessments also had benefits for more experienced markers as it served to remind them of the level of student performance half way through the academic year. Furthermore the formative assessment gave the markers some feedback on their teaching abilities.

### Calibration and the need for rigour in marking

Some markers went to considerable lengths to calibrate themselves. One respondent read all the assessments through to establish a level of student performance before beginning marking. Although experienced markers did use internal calibration as a marking aide, novice markers were using this technique more frequently.

GP4: *Right, I initially went through them all [...] quite quickly. I felt that I had to do that because I'd never read any before and I thought until I've read all 10, I'll then have an idea of what's good, what's in the middle and what's bad *.(new marker)

It was also common for the markers to double mark with themselves as a reliability exercise. Three of the markers described how and why they did this. They felt it improved their fairness, reliability and improved their confidence in their own marking.

Markers described a tension between an instinct to rely on their 'gut-feelings' and their desire to apply the agreed marking schedule in an objective manner. Use of gut-feelings was more often used by experienced markers, because they felt they had more experience on which to base their instinctive impressions. If marks ascertained through gut feelings and through the marking schedule were commensurate, this produced further confidence in their instincts. If there were discrepancies, the marking schedule was considered more trustworthy. The markers who used gut-feelings to pre-classify an assignment felt that this method was productive and accurate, although at the same time, either explicitly or through laughter, they acknowledged it was not the recommended method.

GP6: *I must admit what I tend to do is give one read through first, and have a sort of global sense of where I think that assignment sits and then see if the marking tool actually agrees with the global fit. I suppose in all honesty you don't, I suppose if you're being true to the process you work the other way, ....if its inconsistent with my original view I have to use that then to moderate the mark *.(experienced marker)

GP1: *How I tend to do things partly because of experience and partly because of my style, is to decide on a grade and then work backwards as to how and where to allocate the mark to make that grade in the first place, if that makes any sense. And it was very gratifying to discover that both ways ended up with the same result *.(experienced marker)

The value or otherwise of using this type of global marking was explored during the respondent validation exercise. There was agreement that there was a place for it within marking. It allows markers to award or penalise marks if they believed assessments were either insightful or flawed in some way that could only be judged subjectively, giving them flexibility that is not available within the marking schedule.

GP8: *The marking schedule reduces the spread of grades that you give ....but then we use our gut feeling to make the spread a bit better*.

GP9: *That's it. We use our gut feeling because the marking schedule isn't accurate enough to reflect it*.

Moderator: *So do you think that gut feeling has a place even if you have the perfect marking schedule?*

GP10: *Yes [agreement]*

(respondent validation meeting)

### Role taking

Tutors adopted various roles within the marking process, in relation to each other's marking. The most commonly expressed roles were that of 'hawk' or 'dove' (or hard and soft marker). Another common role was as 'student advocate'. In adopting this identity the markers were trying to empathise with the students' circumstances.

GP1: *I am still conscious of what it was like to be a medical student and how much work was involved [....] and I think we all took a variety of different roles. I mean I found my role was to act to some extent as student advocate *.(experienced marker)

GP5: *I was appalling [as a student]. I didn't work (laughs), and I think I probably would have failed this. Well no, I wouldn't (laughs) but you know, I just think that yes I can see they're my students you know, I can see it in some of them but it's best not to get too personal *.(moderate experience)

A further role was that of 'expert' or 'novice'. Although respecting the autonomy and professionalism of members of the group regardless of experience, there were more confident markers who were willing to take the lead.

### The influence of others

Marking style was influenced by personal contact with other markers. For example, GP7 reflected on his early experiences as a marker and how he had been taught the importance of analytic rigour. Early exposure to different marking styles and approaches through peer review may therefore be very influential.

GP7: *He was a big influence on my style. If it had been another tutor it may have been a completely different influence [...] I think he taught me that I needed to be analytical about it, every paragraph and to make sure I was properly weighing up – that this wasn't some sort of general impression, get a B or an A or a C or you don't like this and there seem to be quite a few omissions so you get a D, but you know you really do need to give some thought to each and every category within the marking criteria*.(moderate experience)

### Difficult decisions

Markers were invited to discuss areas where they faced difficult decisions. These fell into three main categories. The first was making a decision in the middle range of marks, the second was failing a student and the third related to students' poor choice of case.

GP2: *the good ones are really easy to mark and the bad ones are relatively easy to mark. But the ones in the middle I find more difficult to judge because of the marking schedule, it's only out of 20 and there were lots of different sections so you've only got one or two marks per section....you know, judging between say, giving them 2 or 3 marks actually at the end can make a fairly big difference on their overall mark *.(new marker)

New markers found it hard to fail students. Their concerns were whether the mark might be challenged and whether they could be confident in their decision. Some respondents stressed the importance of good note keeping for accountability purposes in case the student appealed against their mark.

GP3: *If the student moaned about what mark I had given them, I would be able to justify it because I've got faith in my own record. I just say well look I'm sorry I didn't think it was a full account. I didn't think that it was a good discussion of this. I didn't – you know. That's what I thought at the time and that's how I've marked it *.(experienced marker)

In some of the interviews it was apparent that the marker felt genuinely upset for the student and that possibly this empathy arose from their own recollections of being an undergraduate medical student.

GP4: *it's hard to send somebody back a fail paper and I didn't really want to. And I was a bit anxious about that sort of – well I did show the other lecturer the fail paper and he agreed, gave them less than I did. So I kind of went over those quite a bit to sort of try and decide whether they would be, you know, worth any more because they obviously write something but somehow they've missed the mark of what it is*.(new marker)

The third area of concern for markers was when they felt students had chosen the 'wrong' case around which to focus their assignment. A wrong case was typically one which led the student to depart from the marking schedule. In some cases the markers felt this may have been because the student had been poorly advised by their tutor. Therefore there were concerns that the marker was making a judgement about the tutor's performance rather than the student's discussion of the patient. They also thought it resulted in extra work for markers in the long run, such as dealing with queries and complaints about the marking process.

### Views about the assessment

The purpose of the long case report is to assess students' ability to construct a diagnostic synthesis of an acute case in Primary Care they have seen themselves. Some markers supported the continuation of the assessment, arguing that it did appear to discriminate well between good and bad students (that is, good students tended to perform well at it and bad students tended to perform less well). Others expressed some doubt about the validity of the assessment, relating this worry to whether it should be used as the sole evaluation of the students' Primary Care experience, and whether it was testing their English rather than their diagnostic skills. It is taken into account in this reported assessment by inclusion of writing style as part of the marking schedule [3].

### Improving the process

During the interviews the respondents also discussed how marking could be made a more formative experience for themselves. Both new markers said that they would like to see the types of feedback comments that others were giving to their students. This was primarily so they could judge whether the amount and type of feedback that they were giving was appropriate. The more experienced markers also reflected that, in the early years, they would have wanted to see examples of failing, moderate and good students' assessments along with the markers comments for each of those assessments.

GP4: *For me I would have found it quite interesting to have had you know good, medium, bad with comments as to what – I mean it would have been very interesting what we comment on....Like I asked this other tutor to send me her comments, she might be commenting on completely different things to myself. I would be very interested to see a selection of other comments *.(new marker)

The interviews resulted in suggestions for improvement: one of the markers, when considering the tight grading of the marking schedule, suggested that it could be widened by marking a case study out of 100 rather than 20 points, and indeed later produced her suggested schedule for the other markers to consider. In addition it was suggested that more effort should be made to inform tutors, both in writing and at their annual conference, about adequate case selection so that students are not penalised for choosing the wrong type of case.

## Discussion

Markers were concerned about their internal and external reliability, and their ability to be objective. The consequence of changes in their own mental perception and performance could mean that students' marks might be affected by the order in which they were marked, a concern that is echoed in a review paper on affective influences on judgements and information processing [8]. The likelihood of being swayed by one's own stylistic preferences in an essay-style assessment is obviously greater than when marking a structured assessment, a point that has also been noted in other types of assessment such as oral examinations [9]. The tension between rewarding sophistication and creativity in writing, whilst ensuring the test is assessing what it aims to test has been described previously in relation to the methodology of designing assessment tools [10].

Internal calibration, that is comparing the piece of work being marked with others of its type, was the most commonly practised marking aide, with nearly all the respondents describing some variation on the technique. Calibrations were made with other scripts, with recollections of assessments submitted in previous years and even with recollections of their own performance as an undergraduate.

Internal reliability appeared to be more important to markers than comparisons with other markers, although the latter were also mentioned in the interviews.

It was interesting that despite awareness of a range of 'hawkish and doveish' behaviour, no respondent was overtly critical about other markers (another interpretation could be that no-one within the group wished to disturb its dynamics by appearing judgemental of others). Marking style was influenced by personal contact with other markers. They were very interested in each others' marking techniques and keen to debate marking decisions. Newer markers were less confident about their ability to mark and give useful feedback, and wanted someone more experienced to 'shadow mark' with them.

All the markers felt they struggled to make marking decisions with assessments falling into the middle range. One explanation is that the University's grading system results in marks being stretched out in the top and bottom grades (an A represents any grade above 70%, but a B represents just 5 marks between 65–69%, and a C just 5 marks between 60–64%). Consequently in the middle range, small differences in marks can make a big impact on the overall grade. Experienced markers used a mixture of gut feelings and reference to the marking schedule to confirm their decision. Other difficulties included marking an assessment down, when it appeared to the marker that the student may have been ill advised in their choice of a case, or the way in which the consultation had been handled. These issues also appear in two earlier studies that explored the reluctance of lecturers to award failing grades to students [11,12]. In these papers, markers felt low grades to be an emotive and personal judgement of the student, reflecting a sense of failure of their teachers as well as the student. The markers in our study felt that it was unfair to penalise the student for the tutor's misunderstandings.

## Conclusion

This qualitative study describes the processes markers of the described general practice case study undergo when preparing to mark written assessments, describing their ideas, concerns and expectations. As the study was being instigated by the department that employed the interviewees, it could be argued that they were unlikely to mention cutting corners in their marking or indeed any other behaviours that might make them appear sloppy. However, the honesty of comments made, and degree of introspection and detail entered into during the interviews implies that the data collected is valid for the assessment being discussed. In addition, respondent validation took place with the whole group of 16 tutors present, and no new or differing views emerged at this session. The main weakness of the study in interpreting the results is that the interview sample size is small, and entirely made up of GP teachers. These tutors have protected time for marking, so it could also be argued that they were able to be more conscientious and careful in their methods of marking. The study is case specific in that it relates to marking just one piece of written work. Marking other types of work, or by tutors from other disciplines who do not have protected time could result in different, more pragmatic approaches to the marking task. Therefore conclusions that can be taken from the data are limited, and may not be capable of generalising to other marking situations, but they give an insight into the needs of both new and experienced markers.

This paper forms part of a larger study [13] to look at the feasibility of peer review of marking in the training and calibration of markers, as suggested by previous research on double marking [4]. As described earlier, it was a small scale, exploratory study, looking at a range of experience in markers and whether experienced markers had different needs or a different approach to inexperienced markers. The study adds to previous knowledge in this field by illustrating the degree of insecurity markers feel when they approach marking a written piece of work, even though they have been supplied with detailed marking guidelines and a comprehensive marking schedule. It appears that this is because they realise the degree of subjective judgement that underlies what seems to be a well-organised process, while being anxious to give as fair a mark as possible for each piece of work. The results also show how markers compensate for this by putting a great deal of effort into the marking, adopting a variety of techniques – calibration, internal double marking, use of 'gut feelings' and informally seeking a second opinion. Some read through papers several times before awarding a mark. Analysis of the data showed that there did not appear to be much difference between markers, regardless of their experience, and all markers were interested in the idea of peer review of marking as a way of decreasing their insecurities about marking a subjective piece of work, and all saw it as a potentially useful idea. We anticipate that the information collected in this paper will be useful for organisers of assessments to plan the likely needs of their markers, and to incorporate relevant training in marking prior to the marking itself. Strategies that could be used include providing a range of worked examples from previous years, training in the use of a standardised marking schedule, and 'shadow marking' with an experienced peer marker.

Markers also take on a variety of marking 'roles' dependent on their personality and their experience. New markers appeared to be influenced by other markers early on in their marking career, and course convenors should bear this in mind if pairing markers for peer review of marking. In particular new markers would benefit from further guidance in their marking, such as examples of feedback and typical assessments that fall into the categories of 'good', 'bad' and 'average'. Wolfe describes a model of the cognitive framework and processes used by markers to show that 'proficient' markers seem able to internalise and use the marking schedule, being more likely to read straight through the essay, withholding judgement until the reading task was finished [1]. There may be tips on marking technique from studies such as these that could be used in training new markers.

Another way of making this preparation more systematic could be through peer review of the marking process. Peer review is an established method for giving feedback and non-threatening quality control in teaching and assessment in a variety of situations [14-17] but its use in review of marking is limited so far. Peer calibration prior to marking a written paper that does not involve meeting and discussing marking interpretation has been described [18,19], but we would advocate face to face structured peer review following the initial marking of a small sample of papers. We have explored this technique [13], and found markers thought it valuable regardless of their level of experience. It improved confidence in their marking abilities, ensured consistency, shared responsibility for failing students, increased awareness of marking style and moderated extreme views. Respondents also commented on how the peer review process could be made more formative for themselves.

Insights from this study may help course convenors to consider training markers, since the anxieties and difficulties faced by our markers are unlikely to be unique. Our larger study, which explored peer review of marking as a way of training and calibrating marking, has also been reported [13]. Our findings may lead to research into better methods of supporting and training markers of assessments with subjective components, including orals, video consultations and written essay work.

## Competing interests

The author(s) declare that they have no competing interests.

## Authors' contributions

KHa conceived the study and obtained funding, all authors contributed to its design, FW collected the data, KHa and FW analysed the data, all authors contributed to the drafting of the manuscript and revising it critically for its intellectual content. All authors have given their approval for the final version to be published.

## Pre-publication history

The pre-publication history for this paper can be accessed here:



## Figures and Tables

**Table 1:** Issues covered in semi-structured interviews:

• Preparations for marking
• Effect of previous experience on marking on the marking process itself
• Opinions of the assessment and its suitability for marking
• Opinions of the marking schedule and how it was used by the markers
• How marking decisions were made, especially difficult decisions
• What could improve the marking process for markers

**Table 2:** Themes drawn from interviews with markers

• Concerns about marking
• Calibration of marking
• Role taking in marking
• Influence of 'others'
• The range of difficult decisions to be made
• Views of the assessment
• Improving the assessment process
